# A danger of low copy numbers for inferring incorrect cooperativity degree

**DOI:** 10.1186/1742-4682-7-40

**Published:** 2010-11-01

**Authors:** Zoran Konkoli

**Affiliations:** 1 Chalmers University of Technology, Department of Microtechnology and Nanoscience, Bionano Systems Laboratory, Sweden

## Abstract

**Background:**

A dose-response curve depicts the fraction of bound proteins as a function of unbound ligands. Dose-response curves are used to measure the cooperativity degree of a ligand binding process. Frequently, the Hill function is used to fit the experimental data. The Hill function is parameterized by the value of the dissociation constant and the Hill coefficient, which describes the cooperativity degree. The use of Hill's model and the Hill function has been heavily criticised in this context, predominantly the assumption that all ligands bind at once, which resulted in further refinements of the model. In this work, the validity of the Hill function has been studied from an entirely different point of view. In the limit of low copy numbers the dynamics of the system becomes noisy. The goal was to asses the validity of the Hill function in this limit, and to see in what ways the effects of the fluctuations change the form of the dose-response curves.

**Results:**

Dose-response curves were computed taking into account effects of fluctuations. The effects of fluctuations were described at the lowest order (the second moment of the particle number distribution) by using the previously developed Pair Approach Reaction Noise EStimator (PARNES) method. The stationary state of the system is described by nine equations with nine unknowns. To obtain fluctuation-corrected dose-response curves the equations have been investigated numerically.

**Conclusions:**

The Hill function cannot describe dose-response curves in a low particle limit. First, dose-response curves are not solely parameterized by the dissociation constant and the Hill coefficient. In general, the shape of a dose-response curve depends on the variables that describe how an experiment (ensemble) is designed. Second, dose-response curves are multi-valued in a rather non-trivial way.

## Background

The Hill function is frequently used to infer the degree of cooperativity of the chemical reaction in which ligand molecules bind to a protein [1]. Often, the binding of a ligand increases the association rate for the binding of the next ligand. Such reactions are said to be (positively) cooperative. There are examples of cooperative reactions in cell biology. The classical example is the binding of oxygen molecules by hemoglobin [1]. Other perhaps less well-known examples would be parts of the Notch signaling and 30 S ribosome assembly processes [2], as well as the assembly of cholesterol-sphingomyelin complexes [3]. Also, the noise characteristics of various ligand binding reactions were studied theoretically in [4] and some of the experimental systems could be classified as cooperative reactions. A cooperative reaction builds a final complex successively. If strong cooperativity is present, the dynamics of the system can be studied using Hill's model, at least to a first approximation [5].

Hill's model is a grossly simplified version of reality. The model is constructed by assuming that binding and unbinding of ligands occur in *one *step as

(1) $$C_{0}+hA\leftarrow C_{h}$$

where *C*_0 _denotes a protein that binds ligands *A*, and *C_h _*is the ligand-protein complex. The Hill coefficient *h *describes the number of binding sites on the protein. Both the forward and the back reactions are allowed.

Strictly speaking, the Hill coefficient in Hill's model (1) is a stoichiometry coefficient and should be an integer number larger than zero. However, in the calculations that follow, *h *will be allowed non-integer values. Thus in the context of this work the Hill model should be understood more from a model average perspective, where the Hill coefficient is an effective parameter.

An important quantity related to Hill's model is the fraction of the proteins that are bound

(2) $$\varphi \equiv \frac{c_{h}}{c_{0}+c_{h}}$$

In particular, the dependence of *φ *on the amount of unbound ligand in the system *a *is of considerable interest, and is referred to as a dose-response curve. A function frequently used to fit a dose-response curve is the expression derived by Hill, the so-called Hill function, given by

(3) $$\varphi_{H}(a)=\frac{\frac{a^{h}}{K}}{1+\frac{a^{h}}{K}}$$

where *c*_0_, *c_h_*, *a *are used to denote the amounts of unbound proteins, bound proteins, and free ligands, respectively. Please note that the Hill function is only parameterized by *K *and *h*. When fitting experimental data to extract *K *and *h*, it is useful to allow *h *to be a real number. Also, the Hill function is used frequently in theoretical studies to model cooperativity effects.

In general, *c*_0_, *c_h _*and *a *can denote average particle numbers, particle concentrations or partial pressures. It really depends on the types of experiments one wishes to describe. The dissociation constant is essentially controlled by the ratio of the forward and the backward reaction rates.

The original Hill's model is unrealistic since a truly multiparticle reaction with a high Hill's coefficient would be a very unlikely reaction event. The probability that all required ligand molecules meet at the right place, at the right time, is very small. The model was already criticised by Hill himself [6,7]. Subsequently, more realistic models were suggested in a series of studies: Adair [8]; Monod, Wyman, Changeux [9]; and Koshland, Nemethy, Filmer [10]. The difference between the models was critically investigated on the mean field level in [5], which confirmed Hill's original claim that the Hill equation can be used in a case of strong cooperativity when intermediate states are short-lived. For a reaction set that appears strongly cooperative as in (1), the Hill coefficient provides a rough measure of the cooperativity degree of the reaction.

Despite the problems discussed above, the use of Hill's model has some merits [1], and the Hill equation is used frequently in many fields as discussed in review article [11]. Accordingly, in this work, Hill's model will be taken as a basic standard for describing multiparticle (cooperative) reactions. The validity of the model has been extensively investigated previously. The conditions for safe usage of Hill's model can be easily verified.

From now on, it will be assumed that the Hill model under investigation is a valid alias for a more complicated multiparticle-like reaction scheme. The focus will be on investigating the correctness of the resulting Hill's function *φ_H_*(*a*) in a low particle number limit. The ultimate goal of this study is to investigate in what ways the effects of the noise related to the low copy numbers affect the form of the dose-response curve predicted by Hill. Please note that such a goal enforces consideration of a closed system. For an open system, where injection and the decay of particles are allowed, one cannot use the Hill function at all.

## Results and discussion

### Model description

The fundamental quantity we wish to understand is the fraction of bound proteins *φ *in a situation when particle numbers are low. This is done by considering a closed system in a well mixed regime. In such a situation it is sufficient to count the particles. In the following, *n*_0_, *n_h_*, and *n_A _*will denote the number of *C*_0_, *C_h_*, and *A *particles respectively. A stochastic model will be considered with the forward reaction rate *α *and the back reaction rate *β*. The rates have the dimension of inverse time. Owing to the stochastic nature of the model, the particle numbers will fluctuate. The ensemble averages of fluctuating quantities will be denoted by ⟨.⟩. Accordingly, particle amounts will be expressed in terms of average particle numbers, *c*_0 _= ⟨*n*_0_⟩, *c_h _*= ⟨*n_h_*⟩, and *a *= ⟨*n_A_*⟩. In such a case the dissociation constant in equation (3) is precisely given by

(4) $$K=\frac{\beta h!}{\alpha }$$

The expression for *K *in (4) can be obtained from the stationary state equations that describe the system in the mean field limit. Use of equations (27-29) and (30) in the methods section leads to the desired result. Strictly speaking, the variable *K *is not a dissociation constant, but it can be related to it by trivial rescaling by the volume of the system.

For any type of initial conditions the dynamical system at hand will reach equilibrium. The focus will be on investigating the equilibrium state of the model, which in turn will enable us to compute the dose response curve *φ *(*a*).

### Analytical description of system is possible

The central technical result of this paper is the derivation of the nine (non-linear) equations (5-13) with nine unknowns. These equations describe the equilibrium state of the model. The derivation of the equations is described in the methods section. The equations can help in analytical understanding of the problem.

The first three stationary state equations are given by

(5) $$Kc_{h}=c_{0}a^{h}\chi_{a0}^{h}\chi_{aa}^{\left(\begin{array}{l} h\\ 2 \end{array}\right)}$$

(6) $$c_{h}+c_{0}=\langle P_{0}\rangle$$

(7) $$a+hc_{h}=\langle L_{0}\rangle$$

In equation (5), and in the following, the symbol *χ *with a subscript denotes a correlation function. Correlation functions were introduced previously (Konkoli, Z.: Multiparticle reaction noise characteristics, submitted) and describe fluctuations. The situation when all *χ *= 1 corresponds to the mean field limit, where the effects of fluctuations are absent. It is easy to see that in such a case equations (5-7) combine to give the classical Hill function in (3). However, the correlation functions do not equal one in general, and the expression for the Hill function in equation (3) might be invalid.

Equations (6) and (7) express the fact that the total number of protein complexes (with and without ligands) *P*_0_, and the total number of ligands in the system (both free and bound) *L*_0_, cannot change over time. Averages ⟨*P*_0_⟩ and ⟨*L*_0_⟩ need to be used; depending on an ensemble, these quantities might be stochastic. It ultimately depends on how the system is prepared during an experiment.

The remaining six equations feature correlation functions heavily. The first three are

(8) $$\chi_{0h}=\chi_{00}\chi_{0a}^{h}$$

(9) $$\chi_{hh}=\chi_{0h}\chi_{ha}^{h}$$

(10) $$\chi_{ha}=\chi_{0a}\chi_{aa}^{h}$$

and are obtained from analysis of the dynamics that brings the systems to a stationary state. The last three equations are the conservation laws that express the fact that initial fluctuations in *P*_0 _and *L*_0 _cannot change over time:

(11) a2χaa+2hachχha+h2ch2χhh=〈L02〉−a−h2ch

(12) c02χ00+2c0chχ0h+ch2χhh=〈P02〉−〈P0〉

(13) c0aχ0a+hc0chχ0h+achχha+hch2χhh=〈L0P0〉−hch

The nine equations with the nine unknowns (5-13) are the central result of the paper. The equations are non-linear and fully describe the stationary state of the system when the effects of particle number fluctuations are taken into account. The observables of interest (average numbers of particles and correlation functions) are implicit functions of the ensemble properties ⟨*P*_0_⟩, ⟨*L*_0_⟩,$\langle P_{0}^{2}\rangle$, $\langle L_{0}^{2}\rangle$, and ⟨*P*_0_*L*_0_⟩.

The equations are not exact. They were derived using the Pair Approach Reaction Noise Estimator (PARNES) method introduced previously (Konkoli, Z.: Multiparticle reaction noise characteristics, submitted). The PARNES method works by approximating higher order moments of a particle number distribution by second order moments. Should the need arise, the method can be easily extended beyond the pair approach level.

The PARNES method is based on the usage of correlation forms. The correlation forms are used in studies of spatially extended diffusion controlled reactions [12]. They are employed to close the hierarchy of many-point density functions. In the present work, the particular methods discussed in [13] were adopted to study a well mixed reaction system. Because a second quantization formalism is used, the PARNES approximation is naturally expressed as a closure relationship for factorial moments of a particle number distribution. The implementation of the closure procedure is shown in the methods section. There are other ways to perform the closure [4,14-18].

Clearly, once moments are given it should be possible to work backwards and extract the form of the particle number distribution function. This is a rather non-trivial problem and will be studied else-where. Essentially, the PARNES approximation is an expansion around the Poisson distribution. For *χ *≈ 1 the distribution function is Poisson-like. Situations with *χ <*1 and *χ >*1 describe sub- and supra-Poisson regimes respectively.

### The Hill equation is valid for large copy numbers

It is possible to see that when particle numbers become large the correlation functions approach the mean field limit in which all correlation functions are equal to one. For example, by neglecting the *a*-*h*^2^*c_h_*, ⟨*P*_0_⟩ and *hc_h _*terms in equations (11), (12) and (13) respectively, and assuming that $\langle L_{0}^{2}\rangle \approx {\langle L_{0}\rangle }^{2}$, $\langle P_{0}^{2}\rangle \approx {\langle P_{0}\rangle }^{2}$ and ⟨*L*_0_*P*_0_⟩ ≈ ⟨*L*_0_⟩⟨*P*_0_⟩, the resulting equations can be solved by the mean field ansatz. This shows that the Hill function can be used in a large particle number limit.

### A danger of inferring an incorrect Hill's coefficient

The issue is whether all solutions of the central equation system are such that *φ *can be expressed solely as a function of *a*. If this is the case then there is only one equation to use, and there should be no ambiguity regarding the proper choice of Hill's coefficient. By inspecting the form of the central equations it can be seen that this is not the case in general. For example, depending on the procedure used to compute the points in the plot that depicts *φ *(*a*), many curves can be obtained. Equivalently, in more technical terms, for a given reaction system, repeating the experiment to determine *φ *(*a*) with different ensemble setups (the ways the system is prepared), one can obtain different curves for *φ *(*a*). Fitting the curves to *φ_H_*(*a*) would result in different Hill's coefficient for each curve. Thus, the fact that the central equations depend on ensemble properties has far reaching consequences when it comes to extracting the correct Hill coefficient from experiments.

### Numerical tests

**Figure 1:**
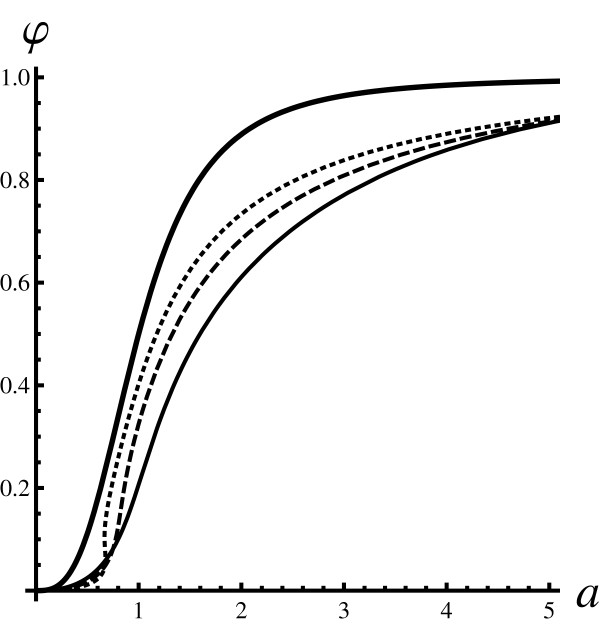
**Fraction of bound proteins (Poisson initial state)**. A dose-response curve (the fraction of the bound proteins *φ *plotted as a function of *a*) for a Poisson-like ensemble: $\langle L_{0}^{2}\rangle$ = ⟨*L*_0_⟩^2 ^+ ⟨*L*_0_⟩ and $\langle P_{0}^{2}\rangle$ = ⟨*P*_0_⟩^2 ^+ ⟨*P*_0_⟩. Each curve is obtained by varying ⟨*L*_0_⟩ for a fixed value of ⟨*P*_0_⟩. The thickest full line is the reference Hill curve *φ_H_*(*a*), plotted with *K *= 1, depicting the mean field limit. The shape of the curve does not depend on the values of the ensemble parameters ⟨*L*_0_⟩ and ⟨*P*_0_⟩. The thin curves are fluctuation-corrected dose-response graphs obtained using the PARNES method. The full line was obtained with ⟨*P*_0_⟩ = 1, the dashed line with ⟨*P*_0_⟩ = 2, and the dotted line with ⟨*P*_0_⟩ = 4. The curves that account for noise (thinner curves) approach the reference mean field curve from below for large values of ⟨*P*_0_⟩ but are distinct otherwise.

**Figure 2:**
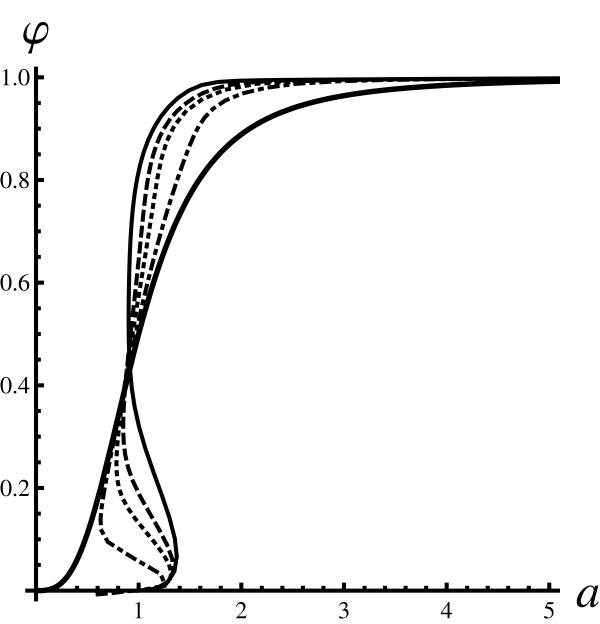
**Fraction of bound proteins (pure initial state)**. Does response curves for the system prepared in a pure state: $\langle L_{0}^{2}\rangle =L_{0}^{2}$ and $\langle P_{0}^{2}\rangle =P_{0}^{2}$ The curves were obtained in the same way as for Fig. 1. The thickest full line is the reference Hill curve obtained with *K *= 1. Other curves describe the effects of fluctuations and were obtained using the PARNES method: the full (*P*_0 _= 2), the dashed (*P*_0 _= 3), the dotted (*P*_0 _= 4), and the dot-dash (*P*_0 _= 8). The thinner curves approach the reference mean field curve for large values of *P*_0_. The curves are distinct and their shape depends on the value of *P*_0_.

The question is how much the effects of noise affect the shape of dose-response curves. To address this question the nine equations were solved numerically for relatively low copy numbers of the protein that binds ligands. Figures 1 and 2 shown that *φ *is not solely a function of *a*, but depends on the characteristics of the ensemble as suggested. The figures describe the Poisson and pure ensembles respectively. The curves in the figures clearly depend on the way that is used to prepare the initial state of the system.

Analysis of both figures shows that for large particle numbers the mean field result (the Hill function) is obtained. This is expected, since the mean field description should be correct for large copy numbers. However, in general, the discrepancy from the mean field case can be significant. For Poisson-like initial conditions the reference curve is approached from below. In the case of pure initial states, the reference curve is approached from above (below) for high (low) values of *a*.

For pure initial states, and in the intermediate regions of *a*, *φ *curves are much steeper that the corresponding Hill function. Please note that the curves for pure states are multi-valued since for a given value of *a *there can be more than one value of *φ *(e.g. all thin curves in Figure 2 for values of *a *slightly greater than one are multi-valued). Similar behaviour is observed for Poisson-like initial states but the onset occurs at smaller values of *a *(e.g. the dotted line in Figure 1). The question is whether such behavior is an artefact of using the PARNES approximation.

**Figure 3:**
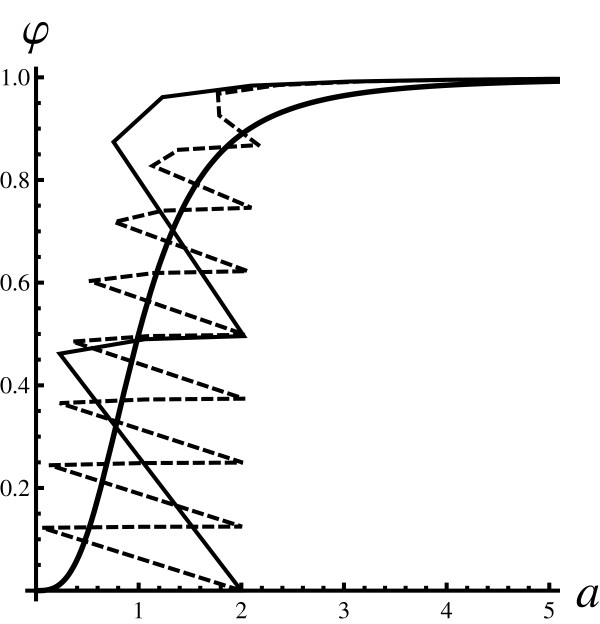
**Fraction of bound proteins (pure initial state), exact result**. Exact dose response curves for a system in pure states. As in Fig. 2 the thickest full line is the reference Hill curve. Thinner curves were generated by direct diagonalisation of the master equation. The thinner full lines are obtained for fixed value of *P*_0 _and looping values of *L*_0_. For each point (*L*_0_, *P*_0_) the master equation was solved numerically and observables of interest were computed. The full line is for *P*_0 _= 2. The dashed line is obtained for a much larger number of receptors *P*_0 _= 8. This figure shows that exact dose response curves are multi-valued. Since not all points are physical, the points were connected using linear interpolation to guide the eye. The dose response curves obtained in such a way are rather erratic. Furthermore, the multi-value character is not an artefact of using linear interpolation. There are many physical points with nearly identical values for *a *having many distinct values for *φ*.

Figure 3 depicts *φ *(*a*) obtained by an exact diagonalisation of the master equation. The figure shows that *φ *(*a*) is indeed multi-valued. The exact solutions exhibit richer behavior than is predicted by the PARNES method. It is very likely that the erratic alternation of points has to do with the fact that not all ligands can be fully absorbed by the receptors. For example, assume that one observes a snapshot of the system dynamics where all proteins in the system have bound all ligands. If one adds more ligands to the system, any number in range from 1 to *h *- 1, exactly that number of ligands will never be bound by the receptor proteins. A similar effect was observed in a related study [19]. Such effects cannot be explained directly by usage of the PARNES method. The PARNES method can describe such behavior only qualitatively.

**Figure 4:**
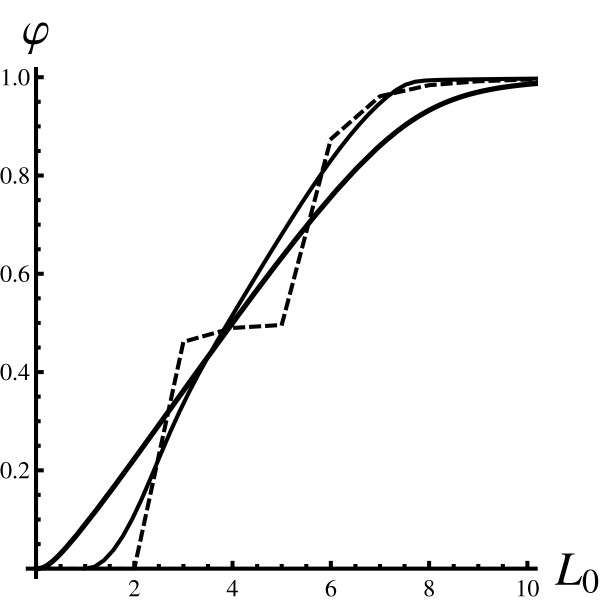
**Fraction of bound proteins; *L*_0 _dependence**. The fraction of the bound proteins *φ *is plotted as the function of free ligands in the system *L*_0 _for the pure state. All curves were obtained for *P*_0 _= 2. The thickest full line is the mean field result. The thinner full line is obtained using the PARNES method. The dashed curve is obtained by exact diagonalisation of the master equation. Please note that the PARNES curve (thin full line) agrees best with the exact result (dashed line).

Figure 4 depicts *φ *as a function of *L*_0 _for a pure ensemble. From a theoretical point of view the dependence of *φ *on *a *is of interest, but *φ *is more likely to be plotted as a function of *L*_0 _in experimental work. Please note that *φ *(*L*_0_) is a single valued function. However, the curve depicting the exact dependence of *φ *on *L*_0 _is not smooth. The notion of the curve is to be understood by interpolating between allowed points since only integer values for *L*_0 _make sense for a pure ensemble. The curve obtained by using the PARNES approximation follows the exact result much more closely than the mean field curve.

## Conclusions

Many dangers have already been recognized in using the Hill function to fit experimental data. The difficulties discussed so far in the literature are mostly related to the fact that the Hill model is only an approximation of a more complicated reaction scheme. This work points to a yet another danger, but in terms of principles.

The findings of this work point to the fact that one should be careful in using the Hill function to fit experimental data when the number of particles in the system is low. The actual dependence of *φ *on *a *is much more complex than predicted by the Hill function *φ_H_*(*a*). First, dose-response curves depend on the way the experiment is done. Repeating the experiment with different ensemble properties could result in a number of distinct curves. Accordingly, equally many values for the Hill coefficient could be extracted. Second, dose-response curves are multivalued in a rather non-trivial way, which has to do with the fact that some ligands will always be unbound, depending on the number of ligands in the system.

The discrepancy between fluctuation-corrected dose-response curves and the Hill function has nothing to with a fundamental flaw in the Hill model itself. The features are rather generic. Similar behaviour is likely to be observed for any more realistic model of ligand binding.

The nine equations obtained in this work could aid experimental studies in which the Hill coefficient is measured. Clearly, to obtain the correct value for the Hill coefficient, one needs to use the correct curve. The nine equations that define dose-response curves could be investigated further to obtain analytical approximations for fluctuation-corrected dose-response curves.

This work can be extended in many ways. The uniqueness conditions for the equations have not been investigated yet. Preliminary numerical investigations show that the structure of the solutions is rather complex, since Mathematica solver had to be fine-tuned to find the solutions. Also, the nine equations allow for non-physical solutions with negative densities or negative correlation functions. This problem can be solved by proper parameterization of the densities. The question is whether some of the features observed here are an artefact of the "all or none" reaction principle that is intrinsic to Hill's model. For example, it is not clear whether the multi-value character of dose response curves will still be observed in more realistic ligand binding models. Some of the issues discussed above will be investigated in forthcoming publications.

## Methods

### Mapping to quantum field theory

The problem at hand is stochastic and can be described by a master equation:

(14) ∂tP(c,t)=α(n0+1)(nA+hh)P(c[+,−,+],t)+β(nh+1)P(c[−,+,−],t)−[αn0(nAh)+βnh]P(c,t)

where ∂*_t _*denotes the time derivative, and *c *= (*n*_0_, *n_h_*, *n_A_*) is a configuration of the system specified by the number of free proteins, ligand protein-complexes and free ligands. The states *c*[+,-, +] and *c*[-,+,-] are defined by

(15) $$c[\pm ,\pm ,\pm ]=(n_{0}\pm 1,n_{h}\pm 1,n_{A}\pm h)$$

where any combination of the plus and the minus signs can be picked at will. The particle number probability distribution function *P*(*c*, *t*) defines the probability that the system is found in a configuration *c *at a time *t*. Please note that the equation contains binomial coefficients that count ways of choosing clusters of *h *particles.

The quantities of interest are observables of the type

(16) $$\langle f(c)\rangle =\sum_{c}f(c)P(c,t)$$

where *f *is an arbitrary function of state *c*. In principle, to compute the averages using (16) is hard. Such a procedure would require the direct solution of the master equation, which is computationally rather demanding. To avoid using equation (16), the equations of motion for the observables of interest will derived. Once in place, these equations of motion can be studied directly. To derive the equations, the problem is mapped on to a quantum field theory using the standard techniques [20]. Thereafter, it is possible to derive the desired equations of motion in a straightforward manner. Please note that any other approach can be used to derive the equations. The filed theory is used in here since it is a useful book-keeping device.

The field theory for the problem is constructed as follows. The particle number probability distribution function is used to construct the generating function

(17) $$|\psi (t)\rangle =\sum_{c}P(c,t)|c\rangle$$

where

(18) $$|c\rangle ={\left({\hat{c}}_{0}^{†}\right)}^{n_{0}}{\left({\hat{c}}_{h}^{†}\right)}^{n_{h}}{\left({\hat{a}}^{†}\right)}^{n_{A}}|0\rangle$$

and the operators in parentheses denote the creation operators for *C*_0_, *C_h _*and *A *particles: ${\hat{c}}_{0}^{†},{\hat{c}}_{h}^{†}$ and *â*^† ^respectively. The operators without the dagger sign, *ĉ*_0_, *ĉ_h _*and *â*, denote the corresponding annihilation operators. The generating function is the linear combination of all possible configurations of the system, where each configuration is weighted by the corresponding probability of occurrence.

The field theory that describes the problem is defined through the expression for the Hamiltonian operator that describes the dynamics:

(19) $$-\partial_{t}|\psi (t)\rangle =\hat{H}|\psi (t)\rangle$$

The requirement for equivalence between equations (14) and (19) fixes the form of the Hamiltonian operator, which turns out to be

(20) $$\hat{H}=\left[{\hat{c}}_{0}^{†}{\left({\hat{a}}^{†}\right)}^{h}-{\hat{c}}_{h}^{†}\right]\left(\frac{\alpha }{h!}{\hat{c}}_{0}{\hat{a}}^{h}-\beta {\hat{c}}_{h}\right)$$

Using quantum field theory formalism, the observable in (16) can be calculated as

(21) $$\langle f\left(n_{0},n_{h},n_{A}\right)\rangle =\langle 1|f\left({\hat{c}}_{0}^{†}{\hat{c}}_{0},{\hat{c}}_{h}^{†}{\hat{c}}_{h},{\hat{a}}^{†}\hat{a}\right)|\psi (t)\rangle$$

where the right hand side of equation (21) is evaluated using the standard commutator rules for the operators

(22) $$\left[{\hat{c}}_{0},{\hat{c}}_{0}^{†}\right]={\hat{c}}_{0}-{\hat{c}}_{0}^{†}{\hat{c}}_{0}=1$$

(23) $$\left[{\hat{c}}_{h},{\hat{c}}_{h}^{†}\right]={\hat{c}}_{h}{\hat{c}}_{h}^{†}-{\hat{c}}_{h}^{†}{\hat{c}}_{h}=1$$

(24) $$\left[\hat{a},{\hat{a}}^{†}\right]=\hat{a}{\hat{a}}^{†}-{\hat{a}}^{†}\hat{a}=1$$

and the fact that

(25) $$\langle 1|=\langle 1|{\hat{c}}_{0}^{†}=\langle 1|{\hat{c}}_{h}^{†}=\langle 1|{\hat{a}}^{†}$$

### Equations of motion

An equation of motion for the observable $\hat{f}$ can be derived from

(26) $$\partial_{t}\langle 1|\hat{f}|\psi (t)\rangle =-\;\langle 1|[f,\hat{H}]|\psi (t)\rangle$$

The equation follows from (19) and the fact that ⟨1|$\hat{H}$ = 0. In the following, to simplify the notation, an expression of the form $\langle 1|\hat{f}|\psi (t)\rangle$ will be abbreviated to $\langle \hat{f}\rangle$. This should cause no confusion between (16) and (21). If the expression contains field theoretic creation and annihilation operators, the expression should be interpreted as in (21).

Using equation (26) with $\hat{f}={\hat{c}}_{0},{\hat{c}}_{h},\hat{a}$ it is possible to derive equations of motion for the average numbers of *C*_0_, *C_h _*and *A *particles given by *c*_0 _= ⟨*ĉ*_0_⟩, *c_h _*= ⟨*ĉ_h_*⟩ and *a *= ⟨*â*⟩. The equations are given by

(27) $$\partial_{t}\langle {\hat{c}}_{0}\rangle =\langle \hat{\Xi }\rangle$$

(28) $$\partial_{t}\langle {\hat{c}}_{h}\rangle =-\langle \hat{\Xi }\rangle$$

(29) $$\partial_{t}\langle \hat{a}\rangle =h\langle \hat{\Xi }\rangle$$

where

(30) $$\hat{\Xi }=\beta {\hat{c}}_{h}-\frac{\alpha }{h!}{\hat{c}}_{0}{\hat{a}}^{h}$$

Please note that the equations contain the expression ⟨*ĉ*_0_*â^h^*⟩, so it appears that we need an equation for that quantity as well. This will be dealt with later.

The fluctuations in the numbers of particles will be described by the second moments of the particle number distribution for all pairs. The equations for the second moments are given by

(31) $$\partial_{t}\langle {\hat{c}}_{0}{\hat{c}}_{0}\rangle =2\langle {\hat{c}}_{0}\Xi \rangle$$

(32) $$\partial_{t}\langle {\hat{c}}_{0}{\hat{c}}_{h}\rangle =\langle ({\hat{c}}_{h}-{\hat{c}}_{0})\Xi \rangle$$

(33) $$\partial_{t}\langle {\hat{c}}_{0}\hat{a}\rangle =\langle (h+\hat{a}\;+h{\hat{c}}_{0})\Xi \rangle$$

(34) $$\partial_{t}\langle {\hat{c}}_{h}{\hat{c}}_{h}\rangle =-2\langle {\hat{c}}_{h}\Xi \rangle$$

(35) $$\partial_{t}\langle {\hat{c}}_{h}\hat{a}\rangle =\langle (h{\hat{c}}_{h}-\hat{a})\Xi \rangle$$

(36) $$\partial_{t}\langle \hat{a}\hat{a}\rangle =\langle [h(h-1)+2h\hat{a}]\Xi \rangle$$

### Conservation laws

The system is closed and five conservation laws can be extracted from the equations of motion. This can be done by taking the appropriate linear combinations of the equations so that the time derivatives vanish. The first two conservation laws are given by

(37) $$\langle {\hat{c}}_{0}+{\hat{c}}_{h}\rangle =\langle P_{0}\rangle$$

(38) $$\langle \hat{a}+h{\hat{c}}_{h}\rangle =\langle L_{0}\rangle$$

and express the fact that the total number of protein complexes (with and without ligands) *P*_0_, and the total number of ligands in the system (both free and bound) *L*_0_, cannot change over time. For example, the first conservation law can be obtained by adding equations (27) and (28).

Related to the two conservation laws discussed above it is possible to derive the three additional laws that describe the conservation of fluctuations in *P*_0 _and *L*_0_:

(39) $$\langle {\hat{a}}^{2}+\hat{a}+2h\hat{a}{\hat{c}}_{h}+h^{2}({\hat{c}}_{h}^{2}+{\hat{c}}_{h})\rangle =\langle L_{0}^{2}\rangle$$

(40) $$\langle {\hat{c}}_{0}^{2}+{\hat{c}}_{0}+2{\hat{c}}_{0}{\hat{c}}_{h}+{\hat{c}}_{h}^{2}+{\hat{c}}_{h}\rangle =\langle P_{0}^{2}\rangle$$

(41) $$\langle {\hat{c}}_{0}\hat{a}+h{\hat{c}}_{0}{\hat{c}}_{h}+\hat{a}{\hat{c}}_{h}+h({\hat{c}}_{h}^{2}+{\hat{c}}_{h})\rangle =\langle P_{0}L_{0}\rangle$$

Please note that the conservation laws involve only quantities that describe the ensemble that was used to prepare the system. The ensemble is defined by five *independent *parameters ⟨*P*_0_⟩, ⟨*L*_0_⟩, $\langle P_{0}^{2}\rangle$, $\langle L_{0}^{2}\rangle$ and ⟨*P*_0_*L*_0_⟩.

### Stationary state equations

The Hill function describes stationary states. Accordingly, the equations of motion will be studied in the long time limit. Requiring that all time derivatives in equations (27-29) and (31-36) vanish gives the set of four equations

(42) $$\langle \hat{\Xi }\rangle =0$$

(43) $$\langle {\hat{c}}_{0}\hat{\Xi }\rangle =0$$

(44) $$\langle {\hat{c}}_{h}\hat{\Xi }\rangle =0$$

(45) $$\langle \hat{a}\hat{\Xi }\rangle =0$$

The equations involve expressions for which additional equations of motion need to be derived. Unfortunately, such a procedure results in an infinite hierarchy of equations. To cut the hierarchy, the PARNES approximation is discussed. In technical terms, all expressions that involve a product of three of more operators are approximated by products of the pair correlation functions. The pair correlation functions are defined as

(46) $$\langle {\hat{c}}_{0}{\hat{c}}_{0}\rangle \equiv \langle {\hat{c}}_{0}\rangle \langle {\hat{c}}_{0}\rangle \chi_{00}$$

(47) $$\langle {\hat{c}}_{0}{\hat{c}}_{h}\rangle \equiv \langle {\hat{c}}_{0}\rangle \langle {\hat{c}}_{h}\rangle \chi_{0h}$$

(48) $$\langle {\hat{c}}_{0}\hat{a}\rangle \equiv \langle {\hat{c}}_{0}\rangle \langle \hat{a}\rangle \chi_{0a}$$

(49) $$\langle {\hat{c}}_{h}{\hat{c}}_{h}\rangle \equiv \langle {\hat{c}}_{h}\rangle \langle {\hat{c}}_{h}\rangle \chi_{hh}$$

(50) $$\langle {\hat{c}}_{h}\hat{a}\rangle \equiv \langle {\hat{c}}_{h}\rangle \langle \hat{a}\rangle \chi_{ha}$$

(51) $$\langle \hat{a}\hat{a}\rangle \equiv \langle \hat{a}\rangle \langle \hat{a}\rangle \chi_{aa}$$

In the strict mathematical sense the PARNES approximation can be expressed as follows

(52) 〈c^0xc^hya^z〉≈〈c^0〉x〈c^h〉y〈a^〉z× ×χ00(2x)χhh(2y)χaa(2z)χ0hxyχ0axzχhayz

where *x*, *y *and *z *are integers greater than or equal to zero. The accuracy of the PARNES approximation has been investigated on a similar model where it was confirmed that it provides a semi-quantitative description (Konkoli, Z.: Multiparticle reaction noise characteristics, submitted). For large particle numbers it is rather accurate. A similar investigation for Hill's model (Figure 4) leads to the same conclusions. The PARNES approximation provides a qualitative description of the stationary state of Hill's model in the low particle number limit.

Finally, using the PARNES method (52), an approximative form of equations (42-45) can be derived. Carrying out the procedure, and combining the result with the conservation laws (37-41), results in the nine equations with nine unknowns listed in (5-13), which were introduced in the results section.

The central equations (5-13) can be obtained roughly as follows. Equation (5) results from the stationary state condition (42), and equations (6-7) are the first two conservation laws (37) and (38) expressed in a new notation. Equations (8-10) result from the stationary state conditions (43-45). Equations (11-13) are derived from the conservation laws for second moments (39-41).

### Numerical recipe

In the general case, the equations are rather involved and cannot be solved analytically. The numerical procedure for solving the equations naturally suggests itself as follows. First, one solves equations (5-7) assuming that all correlation functions are one. This gives the first guess for the average particle numbers *c*_0_, *c_h _*and *a*. The values obtained are inserted into (8-13) to evaluate a guess for the correlation functions. The resulting values can be again used again in (5-7) to obtain even better values for the average particle numbers. The procedure continues until results converge to the fixed point values.

However, the procedure discussed above is numerically unstable in the low particle number limit. The plots in the work were generated by a method similar to the analytic continuation. The equations were solved in the large particle number limit by the method outlined in the previous paragraph, after which the desired point in the ensemble parameter space can be approached incrementally along a line. In every step, the solution from the previous point is used as a guess for the point that follows.

## Competing interests

The author declares that they have no competing interests.
